# Editorial: Advances in radiotherapy for head and neck cancer

**DOI:** 10.3389/fonc.2024.1437237

**Published:** 2024-06-07

**Authors:** Giuseppe Carlo Iorio, Nerina Denaro, Lorenzo Livi, Isacco Desideri, Valerio Nardone, Umberto Ricardi

**Affiliations:** ^1^ Department of Oncology, Radiation Oncology, University of Turin, Turin, Italy; ^2^ Medical Oncology Unit, Fondazione IRCCS Ca’ Granda Ospedale Maggiore Policlinico, Milan, Italy; ^3^ Department of Experimental and Clinical Biomedical Sciences “Mario Serio”, University of Florence, Florence, Italy; ^4^ Department of Precision Medicine, University of Campania “L. Vanvitelli”, Naples, Italy

**Keywords:** HNC (head and neck cancer), radiotherapy, radiation oncology, cancer care, personalized oncology

Modern Radiotherapy (RT) plays a key role in Head and Neck Cancer (HNC). More precise delivery techniques, increasing employment of hadrontherapy, and artificial intelligence (AI) support characterize modern RT, enabling safer treatments with enhanced therapeutic window. Nonetheless, multidisciplinary care paths are necessary to manage HNC patients undergoing RT. This Research Topic features 14 articles exploring the advances in RT for HNC, covering both clinical and technological aspects in this complex oncologic scenario.

The first manuscript by Chen is a remarkable review of one of the topics at the forefront of HNC research: the human papillomavirus (HPV-)related oropharyngeal squamous cell carcinoma RT de-escalation (1). Chen appropriately highlighted how, while enthusiasts argue that the data robustly supports the integration of de-escalation into contemporary practice, skeptics point out that the published data is still relatively preliminary and makes it difficult to make definitive recommendations.

Two manuscripts reported the role of hadrontherapy in HNC (Adeberg et al. and Rampinelli et al.). Hadrontherapy offers advantages in dose distribution and improved radiobiology that may significantly improve the treatment safety and outcomes of certain HNCs (2–4). The retrospective analysis by Adeberg et al. assessed the outcomes and treatment-related toxicity following intensity-modulated RT (IMRT) and a Carbon Ion RT (CIRT) boost for salivary duct carcinoma (SDC). The study showed that multimodal therapy approaches with surgery followed by IMRT and CIRT boost for SDC leads to good local and locoregional disease control. The use of proton therapy reirradiation for HNC is increasing (5). However, reports are heterogeneous, and outcomes can be difficult to interpret (5). Rampinelli et al. assessed outcomes and toxicities of different treatment modalities (including proton therapy) for recurrent nasopharyngeal carcinoma (NPC) in a non-endemic area. A total of 140 patients treated in Italy from 1998 to 2020 were retrospectively assessed. In this series, favorable cases with lower age, comorbidity rate, and stage underwent preferentially endoscopic surgery, as well as patients with shorter disease-free interval from primary treatment. More complex cases underwent re-RT, distributed between photon-based RT and proton therapy. Noteworthy, regarding the intricate context of recurrent NPC, a Chinese multicentre randomized phase 3 trial (published in 2021) demonstrated that endoscopic surgery significantly improved overall survival compared with re-IMRT in patients with resectable local recurrence (6).

Three manuscripts focused on how radiation oncologists should set up multidisciplinary care paths to limit the severity of side effects and encourage tailored monitoring strategies for HNC patients (Santo et al.; He et al.; Patel et al.).

Patients with HNC are at a high risk of malnutrition at the time of diagnosis, and nutritional support or intervention is often needed during and after RT to avoid jeopardizing treatment outcomes (7). Therefore, the role of a multidisciplinary team is to share the outcomes to assess the proper supportive path. The literature overview by Santo et al. highlighted that adequate nutritional screening, assessment, and interventions might increase treatment adherence. Cognitive behavioral therapy (CBT) has already been confirmed to be an effective psychological treatment to avoid or decrease the occurrence of adverse effects in patients with proven malignancies (8). In this regard, the prospective study by He et al. showed that CBT reduced the occurrence, latency, and severity of oral mucositis during chemoradiation therapy for locoregional advanced NPC. A too-often neglected HNC treatment-related toxicity is financial toxicity. Unplanned hospitalizations and emergency department (ED) visits during RT can lead to treatment breaks, leading to a financial burden on patients and the healthcare system (9). Patel et al. retrospectively analyzed the occurrence of ED visits, unplanned hospitalizations, and treatment breaks in HNC patients (n=376) undergoing RT in relation to pain and opioid use as well as other clinical, treatment, and socioeconomic characteristics. The authors found that patients with factors such as heavy opioid use, black race, receipt of concomitant chemotherapy, and lower socioeconomic class may require closer monitoring during RT.

Moving into the “technical” section, this Research Topic is rounded out by two articles (Zong et al.; Cai et al.) focusing on NPC clinical target volume (CTV) optimization and organs-at-risk (OARs) refinement, and a final set of six articles (Luan et al.; Lucido et al.; Gu et al.; Li et al.; Liu et al.; Huang et al.) highlighting advances in machine learning(ML)/deep learning(DL), radiomics and dosiomics.

In terms of modern RT target volumes refinement and optimization for NPC patients, a phase III trial published in 2022 demonstrated that the elective ipsilateral upper-neck irradiation (UNI), sparing the uninvolved lower neck, provided similar regional control and resulted in less toxicity compared with standard whole-neck irradiation (WNI) in patients with N0-N1 disease (10). The retrospective study by Zong et al. aimed to determine the diagnostic value of diffusion-weighted imaging (DWI) and to elucidate the clinical characteristics of medial group retropharyngeal lymph nodes (RLNs) based on multimodal imaging. Additionally, the authors intended to explore the feasibility of optimizing the CTV60 boundary based on the characteristics of medial group RLNs. Among the findings, DWI demonstrated superiority in displaying lymph nodes. Moreover, based on the low incidence of the medial RLNs, CTV60 of medial group RLNs from the skull base to the upper edge of C2 emerged as feasible, leading to dosimetric advantages for protecting swallowing structures. Cai et al. aimed to assess the effects of accessory parotid gland (APG) on the dosimetry of the parotid glands (PGs) during NPC RT and evaluate its predictive value for late xerostomia. Noteworthy, the APG is rarely mentioned in the literature (11). Cai et al. suggested the potential benefits of considering the APG and PGs as a single OAR during RT for NPC. With APG included, the predictive power of the dosimetric parameters for xerostomia tended to improve, although no significant differences were observed. Manual labeling of HN OARs is time-consuming and subjective (12). Therefore, DL segmentation methods have been widely used. However, the accuracy of commercial autosegmentation systems still needs to be evaluated and improved (13).

Luan et al. proposed a parallel network architecture called PCG-Net, which incorporates both convolutional neural networks and a Gate-Axial-Transformer to capture local information and global context effectively. The PCG-Net outperformed other methods, improving the accuracy of HN OARs segmentation and potentially treatment planning for HNC patients. In the setting of using a DL-based autosegmentation model to reduce contouring time without compromising contour accuracy, Lucido et al. conducted a blinded randomized trial employing two HNC expert radiation oncologists and using retrospective de-identified patient data. DL autosegmentation demonstrated significant time-savings for OARs contouring while improving agreement with the institutional gold standard. On the other hand, DL for RT dose prediction has been reported for different tumor sites, but the influence of multiple different levels of input information on the ability to predict dose has not been adequately investigated. A generative adversarial network (GAN) is a class of ML frameworks and a prominent framework for approaching generative AI.

A GAN generates realistic predictions using two concurrent generative and discriminative neural networks (14, 15). Gu et al. used a three-dimensional deep GAN to predict dose distributions for locally advanced HNC RT and achieved results that were highly similar to the clinical plans.

Radiomics is a new research field that decodes tumor phenotypes by quantitatively analyzing image features extracted from medical images, seeking personalized clinical decision-making and better patient stratification (16–20).

Li et al. analyzed the recurrence patterns and reasons in patients with NPC treated with IMRT and investigated the feasibility of radiomics for the analysis of radioresistance. The authors found that radiomic analysis can serve as an imaging biomarker to facilitate early salvage for NPC patients at risk of in-field recurrence. Using methods similar to radiomics, dosiomics analyzes the spatial features of the 3-dimensional patient-specific dose distribution, providing better predictions of radiation-induced results (21, 22). Liu et al. demonstrated that a comprehensive modeling approach combining radiomics, dosiomics and clinical components displayed better predictive values for locoregional recurrence risk following RT than any single factor for locoregionally advanced hypopharyngeal squamous cell carcinoma.

Finally, radiomics and dosiomics can predict treatment-related toxicity for HNC patients. Huang et al. showed that radiomics and dosiomics features (from the planning computed tomography) are correlated with the incidence of grade 4 radiation-induced lymphopenia (G4-RIL) in NPC patients. Noteworthy, RIL has been proven to be a prognostically significant toxicity, affecting survival outcomes in several solid tumors (23, 24). To conclude, this Research Topic presents evidence of the clinical and technological advances in RT for HNC. This broad collection of articles summarizes these exciting active research areas while underscoring the current advances.

## Author contributions

GI: Conceptualization, Investigation, Writing – original draft, Writing – review & editing, Data curation, Methodology, Project administration, Supervision, Validation, Visualization. ND: Conceptualization, Writing – review & editing. LL: Conceptualization, Writing – review & editing. ID: Conceptualization, Writing – review & editing. VN: Conceptualization, Writing – review & editing. UR: Conceptualization, Writing – review & editing.
